# Changes of retinal structure and function in patients with internal carotid artery stenosis

**DOI:** 10.1186/s12886-022-02345-7

**Published:** 2022-03-15

**Authors:** Dong-hui Wu, Lan-ting Wu, Yan-ling Wang, Jia-lin Wang

**Affiliations:** Department of Ophthalmology, Beijing Friendship Hospital, Capital Medical University, Beijing, 100050 China

**Keywords:** Internal carotid artery stenosis, Retinal vascular calibers, Retinal sensitivity, Microperimetry

## Abstract

**Background:**

To investigate the structural and functional changes of the retina in patients with different degrees of internal carotid artery (ICA) stenosis.

**Methods:**

This cross-sectional study included patients with varying degrees ICA stenosis. Clinical characteristics of 41 patients were collected after being divided into four groups according to the ICA stenosis indicated by computed tomographic angiography (Group 0: without ICA stenosis, Group 1: ipsilateral slight ICA stenosis, Group 2: ipsilateral moderate ICA stenosis, Group 3: ipsilateral severe ICA stenosis). Retinal vessel caliber (RVC) was measured quantitatively with the Integrative Vessel Analysis software. The retinal sensitivity was examined with the MP-3 microperimeter. The relationships among central retinal artery equivalent (CRAE), central retinal vein equivalent, arteriole to venule ratio (AVR), mean retinal sensitivity (MS) and ICA stenosis degree were analysed.

**Results:**

The CRAE in Group 3 were significantly smaller compared with Group 0, Group 1 and Group 2 (*P* < 0.001, *P* < 0.001, *P* = 0.002). Significant decrease was found between Group 3 with other groups in MS at fovea (*P* < 0.001, *P* < 0.001, *P* = 0.002). Moreover, there was a positive correlation found between MS and CRAE (Beta = 0.60, *P* < 0.001 at fovea; Beta = 0.64, *P* < 0.001 at 2 degree; Beta = 0.60, P < 0.001 at 4 degree; Beta = 0.55, *P* < 0.001 at 8 degree; Beta = 0.53, *P* < 0.001 at 12 degree).

**Conclusions:**

The present study revealed smaller CRAE and AVR in ipsilateral severe ICA stenosis patients. And the MS decreased in patients with severe ICA stenosis. In addition, MS had a positive correlation with CRAE.

## Background

It is now widely recognized that internal carotid artery (ICA) stenosis may cause severe ischemic cerebrovascular disease. The correlation between asymptomatic ICA stenosis and silent brain infarcts may be related to the degree of stenosis [1]. ICA stenosis can directly affect the eye blood supply, which may cause ocular ischemic syndrome (OIS). OIS is caused by ocular hypoperfusion secondary to stenosis of ipsilateral common carotid or ICA. OIS has a mortality rate of up to 40% within five years of onset [2]. Approximately 66% of the main causes of death are cardiovascular disease, followed by stroke. The most common symptom of OIS is visual loss, some of which, unfortunately, are acute and irreversible. While retinal vascular changes, usually asymptomatic, occur in up to 29% of patients with carotid artery occlusion, and 1.5% progress to symptomatic OIS each year [3]. Lawrence and Oderich noted that the incidence of ocular symptoms increased when ICA stenosis was greater than 50% [4]. Hence, early detection of retinal ischemia is vital for patients with ICA stenosis.

The retina vasculature provides a unique window where features of the systemic microcirculation can be observed noninvasively. With the progress of fundus photography and computer software, quantitative measurement of retinal vessels is widely used in the prediction of cardio-cerebrovascular diseases [5–7]. Current studies have found that ICA stenosis is related to retinal vein diameter widening [8], while there was study also suggested that no correlation existed [9]. In view of this inconsistency, we divided ICA stenosis into different degrees to observe retinal vascular structural changes.

Microperimetry (MP), or fundus-controlled perimetry, is a simple, noninvasive, and rapid visual function test that provides a functional evaluation of retinal sensitivity. Study has shown that MP is even more sensitive than multifocal electroretinography to detect early changes in retinal function [10]. In addition, Machalińska has revealed the influence of ICA stenosis on retinal function [11]. However, MP has not been used in evaluating the relationship between ICA stenosis and retinal sensitivity. For this reason, we observed characteristics in retinal structure at different degrees of ICA stenosis, and used MP to evaluate changes in retinal function, which may provide valuable information to further understand visual performance in ICA stenosis.

## Methods

This cross-sectional study included patients who underwent head and neck CT angiography (CTA) with ICA stenosis at Beijing Friendship Hospital, China, from October 2018 to October 2019. Each participant underwent a detailed ophthalmic examination, including best-corrected visual acuity (BCVA), slit-lamp examination, fundus photography, intraocular pressure (IOP, by noncontact tonometry), and optical coherence tomography (Heidelberg Engineering, GmbH, Heidelberg, Germany). Participants with: a) any optic nerve, orbital or intracranial disease; b) presence of macular disease, cataract with visual impairment, ocular trauma infection; c) amblyopia, strabismus or severe dry eye; d) medication that affect visual field sensitivity were excluded from the study. All participants provided written informed consent.

Participants were divided into 4 groups: without ICA stenosis (Group 0), ipsilateral slight ICA stenosis (Group 1), ipsilateral moderate ICA stenosis (Group 2), and ipsilateral severe ICA stenosis (Group 3). The stenosis degree was evaluated by the NASCET (North American Symptomatic Carotid Endarterectomy Trial) stenosis grading method. According to this method, the vascular stenosis degree (%) = (1 − N/D) × 100%. D represents the normal vessel diameter, and N represents the narrow vessel diameter in this formula. Classification of stenosis degree: no stenosis (stenosis rate is 0), mild stenosis (stenosis rate ≤ 29%), moderate stenosis (stenosis rate 30%-69%), severe stenosis (stenosis rate 70%-99%) and complete occlusion [12]. Baseline data were collected after patients: general information (age, sex); health condition (hypertension, hyperlipemia, diabetes, coronary artery disease, stroke, etc.); daily habits (drinking, smoking).

### Computed tomography angiography examination

A 64-row multidetector CT scanner (Light speed VCT, GE Medical Systems, USA) were used to perform the head and neck CTA. Scans extended from the arch of the aorta to the base of the skull. Injection of contrast medium 65 ml (Iohexol, 300 mg iodine/mL), through the 18 gauge-needle by a power syringe at a rate of 4 ml/s into the antecubital vein. CT parameters were as follows: pixel spacing 0.625 mm, image resolution 512*512, layer spacing 0.8 mm, machine rack rotation speed 39.37 mm/rotation, rack rotation time 0.5 s/rotation, pitch 0.984.

### Retinal vessel caliber measurement

Digital fundus photographs of all patients were obtained by a 45degree high resolution fundus camera (Kowa, Tokyo, Japan). Retinal vessel caliber (RVC) was measured by the Integrative Vessel Analysis (IVAN software, Australia).

**Fig. 1:**
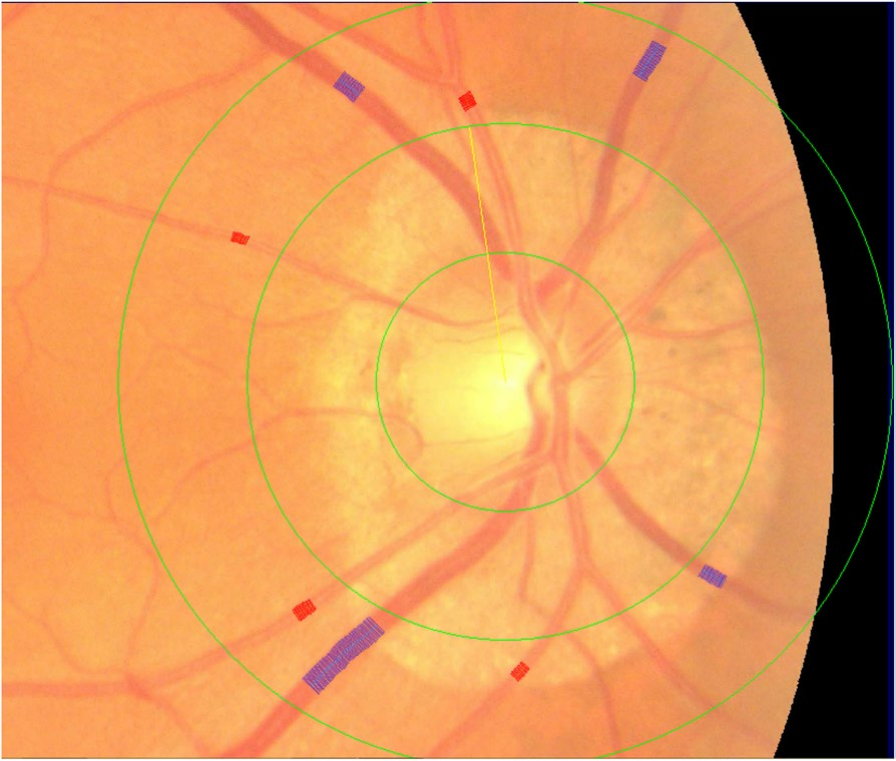
Quantitative analysis of retinal vessel caliber by Integrative Vessel Analysis (IVAN software, Australia). Veins are identified in purple and arteries in red.

On fundus photographs, diameters of the 6 larger branches of the retinal arteriovenous canal were obtained from the area which ranges 0.5–1.0disc diameter distance from the edge of optic disc (Fig. 1). Then we used the modified Parr-Hubbard formula proposed by Knudtson in 2003 [13], which calculate the relationship between branches and trunks of retinal artery, to obtain the following main parameters: central retinal artery equivalent (CRAE), central retinal vein equivalent (CRVE), and arteriole to venule ratio (AVR).

### MP-3 measurement

**Fig. 2:**
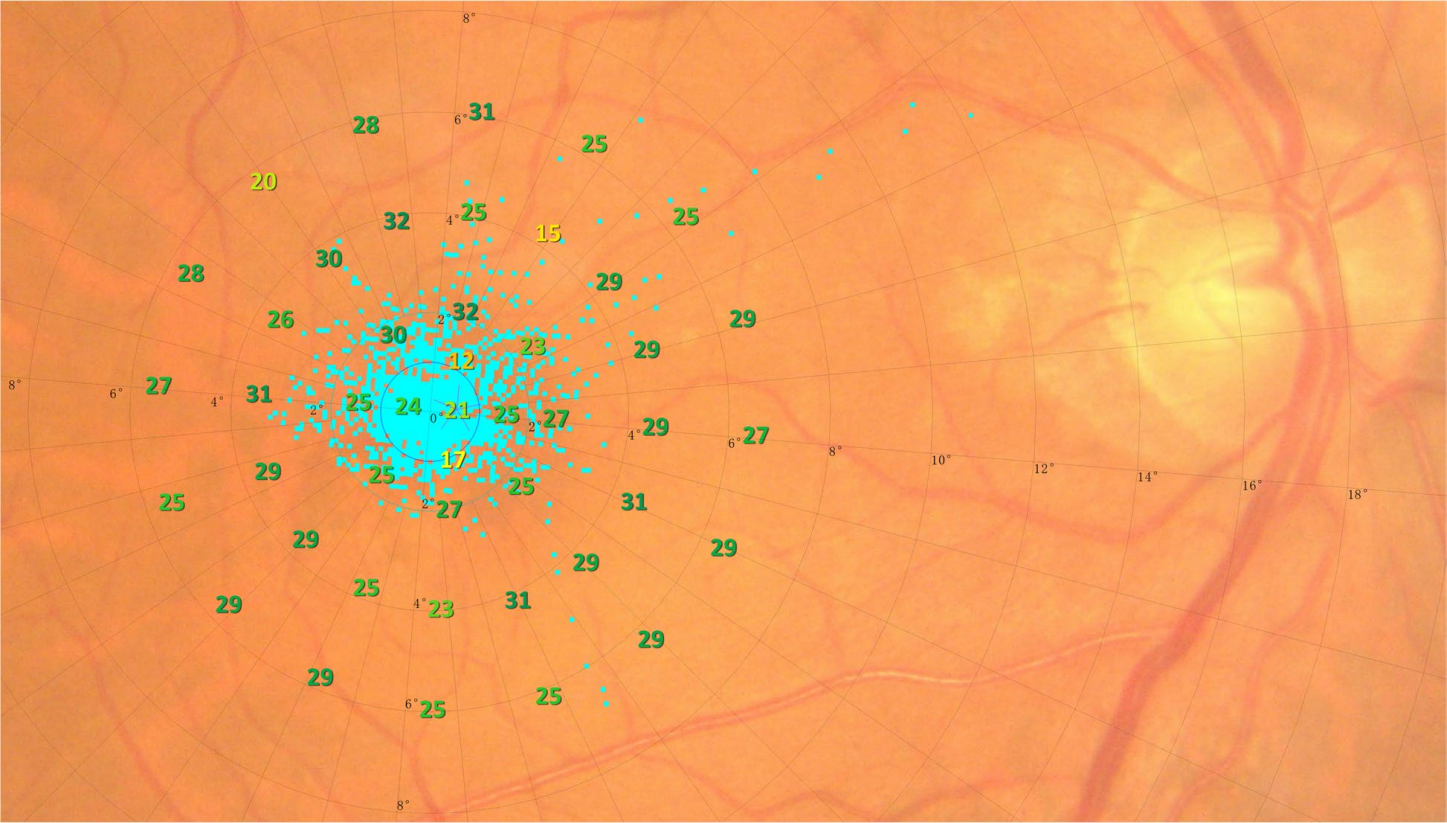
The retinal sensitivity of a representative case (69 years old, male) with internal carotid artery stenosis examined with MP-3 microperimeter (Nidek CO., LTD, Aichi, Japan)

The retinal sensitivity was measured by the MP-3 microperimeter (Nidek CO., LTD, Aichi, Japan), a currently newer commercially available device. And mean retinal sensitivity (MS) is a valuable complementary measurement to visual acuity. MP measures retinal sensitivity based on the minimum light intensity the patient can perceive when a spot of light stimulates a particular area of the retina [14]. Compared with conventional perimetry, MP projected the light stimuli onto the retina directly while automatically tracking fundus at the same time, which helps to accurately measure the sensitivity of specific retinal points [15]. Its measurements were similar to the Humphrey Field Analyzer. The dynamic range of MP-3 stimulation was 34 dB. Background brightness was 31.4 asb, and the maximum brightness was 10,000 asb. MP-3 measured 45 testing sites within 12 degrees of the central retinal diameter using the standard Goldmann III stimulus size (Fig. 2).

### Statistical analysis

Normality tests were performed on all data from each group. The statistical analysis was conducted by one-way analysis of variance for normally distributed continuous variables. Categorical variables were expressed in numbers and percentages and analysed by the χ^2^ test or Fisher’s exact test, as appropriate. Using Bonferroni correction for multiple comparisons. Linear regression was conducted to estimate linear relationships between continuous variables. We also used Spearman rank correlation to explore the relationship between CRAE and ICA stenosis. Statistical analyses were computed by SPSS software (version 26.0; SPSS Inc., Chicago, DE, USA).

## Results

### Demographics

All participants underwent CTA examination and were evaluated by two experienced ophthalmologists. The eye ipsilateral to the ICA was selected for each participant, and a total of 41 participants were included. Based on the degree of ICA stenosis, participants were divided into the following four groups. Demographics of these participants are shown in Table 1. Significant difference was found between Group3 and Group1 (*P* = 0.002) in BCVA. There were no significant differences in age, sex, IOP, hypertension, hyperlipemia, diabetes, coronary artery disease, heart disease, stroke, drinking and smoking.

**Table 1:** Demographic characteristics of the participants

	Group0 (*n* = 11)	Group1 (*n* = 13)	Group2 (*n* = 9)	Group3 (*n* = 8)	*P* value
Age (year)	61.09 ± 3.05	62.31 ± 2.96	63.44 ± 4.07	65.13 ± 1.96	0.051
Sex (male/female)	3/8	6/7	8/1	4/4	0.051
BCVA (logMAR)	0.08	0.02	0.10	0.25	0.004 ^a^
IOP (mmHg)	15.97	15.43	16.03	14.07	0.508
Hypertension (n)	5	9	6	4	0.599
Hyperlipemia (n)	5	10	7	6	0.303
Diabetes (n)	4	7	5	4	0.803
CAD (n)	1	2	1	1	0.989
Heart disease (n)	2	1	1	1	0.891
Stroke (n)	1	2	1	1	0.989
Drinking (n)	6	5	5	3	0.758
Smoking (n)	5	6	5	3	0.905

*BCVA* Best-Corrected Visual Acuity, *IOP* Intraocular Pressure, *CAD* Coronary Artery Disease

^a^ Significant difference between Group3 and Group1 (*P* = 0.002)

### Retinal vessel caliber

**Fig. 3:**
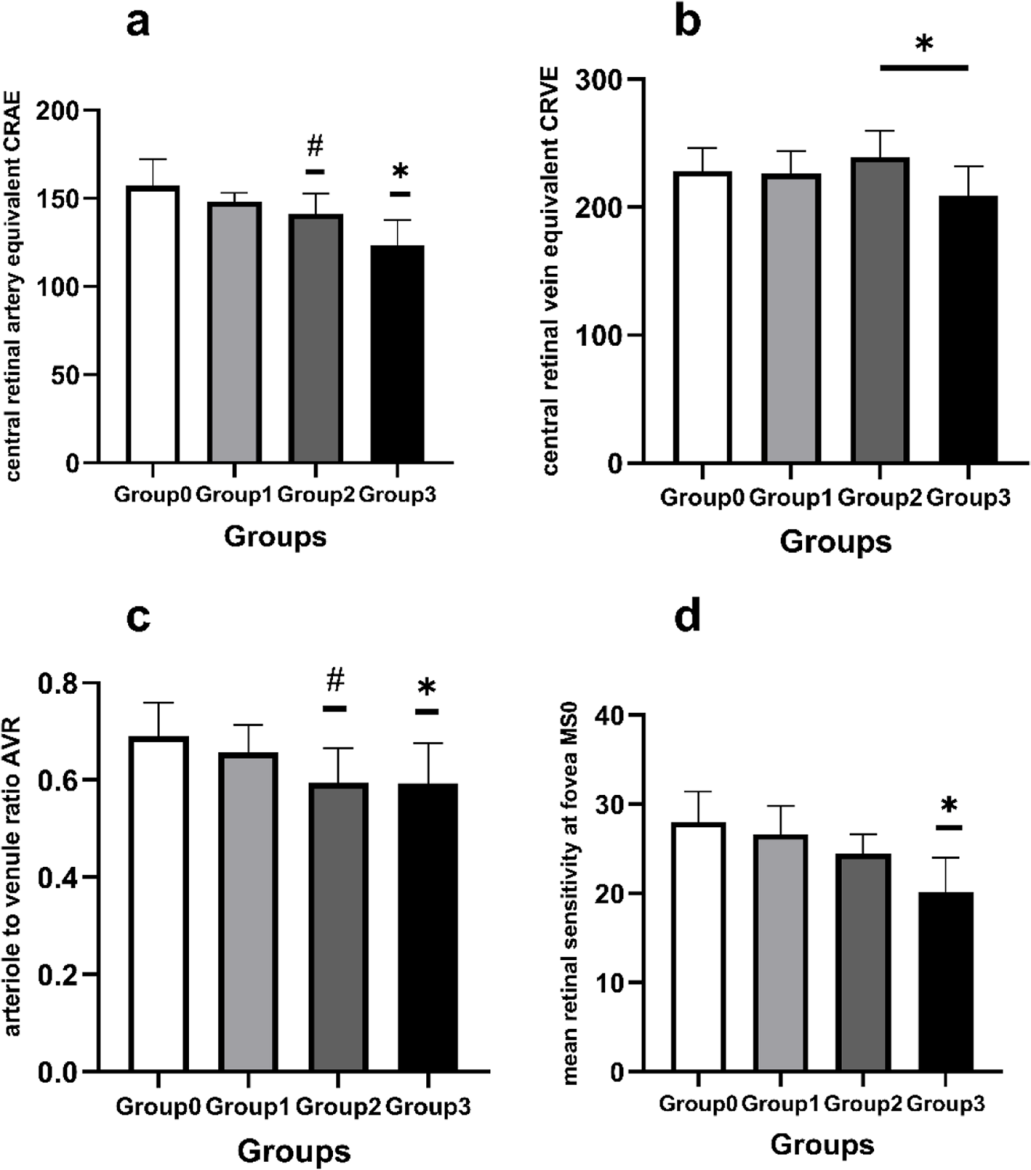
**a**. Comparison of central retinal arteriolar equivalent (CRAE) between groups. *Significant differences between Group3 and the others in CRAE. #Significant difference between Group2 and Group0 in CRAE; **b**. Comparison of central retinal venular equivalent (CRVE) between groups. *Significant difference between Group3 and Group2 in CRVE; **c**. Comparison of arteriovenous ratio (AVR) between groups. #Significant differences between Group2 and Group0 in AVR; * Significant differences between Group2 and Group0 in AVR; **d**. Comparison of mean retinal sensitivity at fovea (MS0) between groups. *Significant difference between Group3 and other groups in MS0; Group0, Group1, Group2 and Group3 respectively refer to groups of patients without stenosis, and with slight, moderate and severe stenosis of ICA

The mean CRAE were shown respectively in Table 2. CRAE were significantly smaller in Group3 compared with other groups (*P* < 0.001, *P* < 0.001, *P* = 0.002), and Group2 was also smaller than Group0 significantly (*P* = 0.006, Fig. 3a). Significant difference was also found between Group3 and Group2 in CRVE (*P* = 0.004, Fig. 3b). AVR represents ratio of retinal arteriovenous diameter. And there were significant differences between Group3 and Group0, Group2 and Group0 (*P* = 0.002, *P* = 0.001, Fig. 3c) in AVR. No significant differences were found between the other unspecified groups. We explored the relationship between CRAE and the degrees of ICA stenosis (rank correlation coefficient = -0.722, *P* < 0.001). CRAE is negatively correlated to the degrees of ICA stenosis. The relationship between CRVE and degrees of ICA stenosis (rank correlation coefficient = -0.160, *p* = 0.318). CRVE is not rankly correlated to the degrees of ICA stenosis.

**Table 2:** Retinal vessel caliber characteristics of the participants

	Group0 (*n* = 11)	Group1 (*n* = 13)	Group2 (*n* = 9)	Group3 (*n* = 8)	*P* value
CRAE (mm)	156.30 ± 14.68	148.18 ± 4.88	141.32 ± 11.40	123.39 ± 13.72	< 0.001 ^a^
CRVE (mm)	226.21 ± 18.55	226.48 ± 17.74	239.35 ± 20.37	211.47 ± 22.42	0.039 ^b^
AVR	0.69 ± 0.07	0.66 ± 0.06	0.59 ± 0.07	0.59 ± 0.08	0.002 ^c^

*CRAE* Central Retinal Artery Equivalent, *CRVE* Central Retinal Vein Equivalent, *AVR* Arteriole to Venule Ratio

^a^ Significant difference between Group3 and other groups (*P* < 0.001, *P* < 0.001, *P* = 0.002), Group2 was also smaller than Group0 significantly (*P* = 0.006);

^b^ Significant difference between Group3 and Group2 in CRVE (*P* = 0.004);

^c^ Significant differences between Group3 and Group0, Group2 and Group0 (*P* = 0.002, *P* = 0.001) in AVR

### Microperimetry

Microperimetry characteristics of the participants are listed in Table 3. Significant decrease was found between Group3 with other groups in MS0 (MS at fovea) (*P* < 0.001, *P* < 0.001, *P* = 0.002, Fig. 3d). Similarly, within MS at 2, 4, 8 and 12 degrees, significant differences between Group3 and the others were also found (*P* < 0.001, *P* < 0.001, *P* = 0.008, at 2 degree; *P* < 0.001, *P* < 0.001, *P* < 0.001, at 4, 8 and 12 degrees). In addition, there was a significant difference between Group2 and Group0 in MS within 2, 4, 8 and 12 degrees (*P* = 0.003, *P* = 0.002, *P* = 0.008, *P* = 0.008). Thus, a significant reduction in MS appears in patients with moderate to severe ICA stenosis.

**Table 3:** Microperimetry characteristics of the participants

	Group0 (*n* = 11)	Group1 (*n* = 13)	Group2 (*n* = 9)	Group3 (*n* = 8)	*P* value
MS0 (dB)	28.00 ± 3.44	26.62 ± 3.23	24.44 ± 2.19	20.13 ± 3.91	< 0.001 ^a^
MS2 (dB)	27.30 ± 2.06	25.94 ± 1.60	24.18 ± 2.95	21.24 ± 2.33	< 0.001 ^b^
MS4 (dB)	28.80 ± 1.10	27.82 ± 1.45	26.24 ± 2.07	22.59 ± 2.36	< 0.001 ^c^
MS8 (dB)	29.67 ± 1.04	28.58 ± 1.32	27.31 ± 1.94	23.47 ± 3.12	< 0.001 ^d^
MS12 (dB)	29.63 ± 1.09	28.49 ± 1.36	27.23 ± 1.78	23.58 ± 3.25	< 0.001 ^e^

*MS* Mean retinal Sensitivity, *MS0* MS at fovea, *MS2* MS at 2 degrees, *MS4* at 4 degrees, *MS8* at 8 degrees, *MS12* at 12 degrees

^a^ Significant decrease was found between Group3 with other groups in MS0 (*P* < 0.001, *P* < 0.001, *P* = 0.002);

^b^ Significant decrease was found between Group3 with other groups in MS2(*P* < 0.001, *P* < 0.001, *P* = 0.008), and a significant difference between Group2 and Group0 (*P* = 0.003);

^c^ Significant decrease was found between Group3 with other groups in MS4 (*P* < 0.001*, P* < 0.001*, P* < 0.001), and a significant difference between Group2 and Group0 (*P* = 0.002);

^d^ Significant decrease was found between Group3 with other groups in MS8 (*P* < 0.001*, P* < 0.001*, P* < 0.001), and a significant difference between Group2 and Group0 (*P* = 0.008);

^e^ Significant decrease was found between Group3 with other groups in MS12 (*P* < 0.001*, P* < 0.001*, P* < 0.001), and a significant difference between Group2 and Group0 (*P* = 0.008)

### Association between mean retinal sensitivity and retinal vessel caliber

Table 4 shows the linear regression analysis performed between MS and CRAE. MS was shown to be positively correlated with CRAE (Beta = 0.60, *P* < 0.001 at fovea; Beta = 0.64, *P* < 0.001 at 2 degree; Beta = 0.60, *P* < 0.001 at 4 degree; Beta = 0.55, *P* < 0.001 at 8 degree; Beta = 0.53, *P* < 0.001 at 12 degree). The regression coefficient of MS0, MS2 and MS4 were larger than others, suggesting that MS had a closer relationship with CRAE at fovea, 2 and 4 degrees. Table 5 shows the linear regression analysis performed between MS and CRVE, indicating that MS had a relationship with CRVE at 4, 8 and 12 degrees.

**Table 4:** Association between mean retinal sensitivity and central retinal artery equivalent

	Standardized coefficients	95.0% CI for B		*P* value
	Beta	Lower bound	Upper bound	
MS0	0.60	0.10	0.25	< 0.001
MS2	0.64	0.08	0.17	< 0.001
MS4	0.60	0.06	0.15	< 0.001
MS8	0.55	0.05	0.15	< 0.001
MS12	0.53	0.05	0.14	< 0.001

*CI* Confidence Interval, *MS* Mean retinal Sensitivity, *MS0* MS at fovea, *MS2* MS at 2 degrees, *MS4* at 4 degrees, *MS8* at 8 degrees, *MS12* at 12 degrees

**Table 5:** Association between mean retinal sensitivity and central retinal venular equivalent

	Standardized coefficients	95.0% CI for B		*P* value
	Beta	Lower bound	Upper bound	
MS0	0.15	-0.72	2.00	0.350
MS2	0.24	-0.42	3.74	0.115
MS4	0.35	0.42	4.76	0.021^a^
MS8	0.39	0.70	4.86	0.010 ^a^
MS12	0.37	0.57	4.81	0.014 ^a^

*CI* Confidence Interval, *MS* Mean retinal Sensitivity, *MS0* MS at fovea, *MS2* MS at 2 degrees, *MS4* at 4 degrees, *MS8* at 8 degrees *MS12* at 12 degrees

^a^ Statistically significant

## Discussion

The relationship between ICA stenosis and retinal sensitivity was analysed for the first time using microperimetry. We found that as the degree of ICA stenosis increased, the MS decreased gradually. The MS at fovea and within 2, 4, 8 and 12 degrees was significantly lower in severe ICA stenosis group than that in other groups. We analysed that severe ICA stenosis can reduce the blood supply of central retinal artery and choroidal vessels in the eye, causing apoptosis of retinal ganglion cells, cone cells and rod cells, resulting in decreased MS. Normally, the central retinal artery is the only arterial blood supply to the inner retina. Our previous research results showed that when the common carotid artery was ligated in rats, the diameter of retinal artery and vein decreased, and the blood flow of central retinal artery decreased significantly, resulting in inner retinal ischemia and ganglion cell apoptosis [16–18]. Blockage of the common carotid artery affects the choroidal circulation, which nourishes photoreceptors, and eventually affects photoreceptors [19]. Palmhof et al. found that RGCs, cone bipolar and cone photoreceptor cells were lost shortly after ischemia induction [20]. Cone photoreceptor cells are strongly affected by ischemic injury. Machalińska’s results showed that the rod mediated response was mainly affected by carotid artery stenosis [21]. Photoreceptoral responses, rod and cone bipolar cell responses were reduced by the ischemia/reperfusion insult [22]. In this study, the MS in the range of 2, 4, 8 and 12 degrees in the moderate ICA stenosis group was lower than that in the non-stenosis group, but there was no difference in the macular fovea between the moderate ICA stenosis group and the non-stenosis group. The reason may be related to the larger number of cones in the macular fovea. The MS in the macular fovea decreased when the ICA stenosis was moderate, but there was no statistical difference. However, when the ICA stenosis was severe, the MS of the macular fovea decreased [23]. Kofoed’s study shows that the retinal cone pathway is affected prior to the onset of clinical eye disease, which corresponds to similar defects in the rod system found by dark adaptometry [24]. Therefore, microperimetry can detect the impaired MS early before the appearance of clinical symptoms.

The results of this study indicate that CRAE decreases gradually as the degree of ICA stenosis increases. Significant differences were found in CRAE between severe ICA stenosis group and other groups, moderate ICA stenosis group and non-stenosis group. This result is consistent with the change of MS with ICA stenosis. We analyse that the possible reason is that insufficient blood supply of ophthalmic artery caused by ICA stenosis caused decrease of retinal blood perfusion and decrease of perfusion pressure, leading to retinal artery contraction, then apoptosis of ganglion cells and cone and rod cells sensitive to ischemic injury, finally resulting in decreased MS. Previous study has shown that ICA stenosis can lead to insufficient blood supply to the ophthalmic artery [25]. Furthermore, bilateral common carotid artery occlusion model rats showed the perfusion of the eyes continued to reduced [26]. The macular perfusion velocities were markedly reduced in OIS eyes compared with unaffected fellow eyes and healthy controls [27]. Retinal blood vessels are auto-regulated. When ocular perfusion pressure is reduced, the retinal arterioles dilate first to maintain constant blood flow and retinal perfusion. Nevertheless, the retinal arterioles contract once the decrease in ocular perfusion pressure exceeds the compensatory limit of retinal vessels [28].

We found that the CRVE increased gradually in non-stenosis group, mild ICA stenosis group and moderate ICA stenosis group, but no significant differences. The CRVE of severe ICA stenosis group was lower than that of other groups, and differences were found between severe ICA stenosis group and moderate ICA stenosis group. We analysed that the possible reason is the contraction of retinal arterioles caused by ICA stenosis resulting in the decrease of oxygen saturation of arterioles, which induces the endothelial dysfunction of retinal veins, leading to the dilatation of retinal veins. The CRVE of severe ICA stenosis group was smaller than that of other groups. We speculated that it may be due to the significant decrease of blood flow in retinal arteriovenous system, resulting in vein contraction and thus a decrease in diameter of retinal vein in severe ICA stenosis group.

The AVR in severe and moderate ICA stenosis groups were smaller than non-stenosis group. This may be related to the decrease of CRAE in severe ICA stenosis group and the increase of CRVE in moderate ICA stenosis group. There was no correlation between CRVE or AVR and the degrees of ICA stenosis. This may be related to the fact that the diameter of retinal vein is affected by many factors. In an Asian population study, a smaller diameter of the retinal arterioles was associated with hypertension, while a larger caliber of retinal arterioles was associated with smoking, dyslipidemia, hyperglycemia, and higher body mass index [29].

Our study found that there was a positive correlation between MS and CRAE. The regression coefficient of MS0, 2 and 4 is larger than the others, suggesting that MS had a closer relationship with CRAE at macular fovea. Our findings may have important implications for discovering ocular ischemic diseases using microperimetry in an early stage. Considerably more work will need to be done to determine the association between MS and CRAE.

There were some limitations to this study. Preliminary results were discussed, since we recruited a relatively small sample of patients. Future studies will require larger sample sizes and longer follow-up periods. Additionally, we will acquire the hemodynamic characteristics of ICA, such as through computational fluid dynamics numerical simulation or transcranial ultrasound Doppler examination. At the same time, we will also collect and analyze the characteristics of the ophthalmic artery, due to the possibility of reflux.

## Conclusions

Microperimetry can provide functional evaluation of retina and detect the impaired MS early before the appearance of clinical symptoms, while the measurement of RVC allows us to obtain structural information. The present study revealed smaller CRAE and AVR in patients with ipsilateral severe ICA stenosis. We also found that the MS decreased in severe ICA stenosis patients. Moreover, there was a positive correlation between MS and CRAE. Future studies with a larger sample size are needed to further verify and explore the relationship between ICA stenosis degree and retinal morphology and function.

## Data Availability

The datasets used and analysed during the current study are available from the corresponding author on reasonable request.

## Abbreviations

ICA: Internal carotid artery
OIS: Ocular ischemic syndrome
MP: Microperimetry
CTA: Computed tomographic angiography
BCVA: Best-corrected visual acuity
IOP: Intraocular pressure
RVC: Retinal vessel caliber
IVAN: Integrative Vessel Analysis
CRAE: Central retinal artery equivalent
CRVE: Central retinal vein equivalent
AVR: Arteriole to venule ratio
MS: Mean retinal sensitivity
